# A kiss to set the rhythm

**DOI:** 10.7554/eLife.19823

**Published:** 2016-08-25

**Authors:** Sonal Shruti, Vincent Prevot

**Affiliations:** 1 Laboratory of Development and Plasticity of the Neuroendocrine Brain and the NEUROBESE International Associated Laboratory, Institut National de la Santé et de la Recherche Médicale, Université de Lille, Unité Mixte de Recherche 1172, Lille, Francesonal.shruti@inserm.fr; 1 Laboratory of Development and Plasticity of the Neuroendocrine Brain and the NEUROBESE International Associated Laboratory, Institut National de la Santé et de la Recherche Médicale, Université de Lille, Unité Mixte de Recherche 1172, Lille, Francevincent.prevot@inserm.fr

**Keywords:** Neuropeptide, Slow EPSP, Synchronization, Neurokinin B, Dynorphin, GnRH, Mouse

## Abstract

Groups of neurons in the hypothalamus synchronize their activity to trigger the production of hormones that sustain fertility.

**Related research article** Qiu J, Nestor CC, Zhang C, Padilla SL, Palmiter RD, Kelly MJ, Rønnekleiv OK. 2016. High frequency stimulation-induced peptide release synchronizes arcuate kisspeptin neurons and excites GnRH neurons. *eLife* **5**:e16246. doi: 10.7554/eLife.16246

**Image** Kisspeptin neurons on both sides of the brain are connected to each other

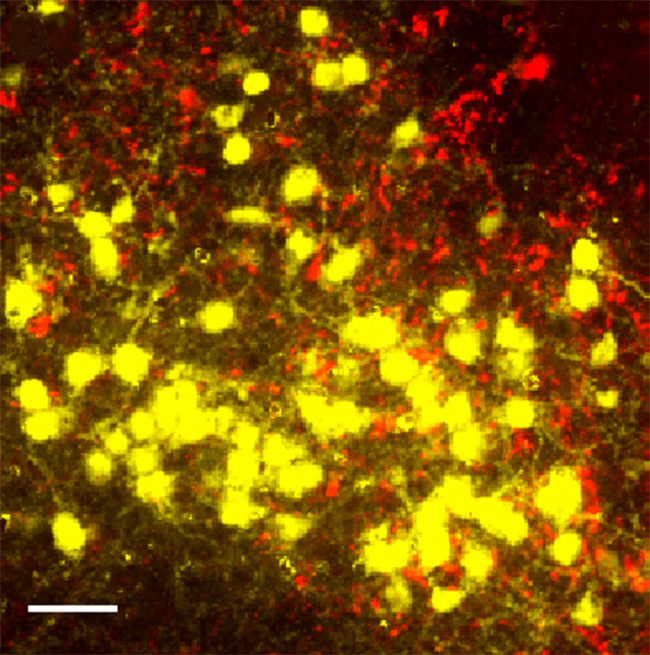

In animals, fertility and reproduction are highly regulated processes that depend on several hormones interacting in a strictly choreographed and rhythmic manner. This regulation starts a long time before birth and is maintained throughout the life of an individual, even after they are no longer fertile (Boehm et al., 2015; Clarke and Dhillo, 2016). However, the system also needs to flexibly adjust to changes including pregnancy, ageing and the availability of food. How is this complex and intricate balance of hormones maintained in the body?

Gonadotropin releasing hormone (GnRH) is the main regulator of fertility and is produced by a group of neurons in a region of the brain called the hypothalamus. This hormone causes cells in the pituitary gland to release several other hormones that regulate the production of sex cells and sex hormones in the gonads. In turn, the sex hormones can also affect the release of GnRH and some pituitary hormones (Cimino et al., 2016; Herbison, 2016).

GnRH is generally released from the hypothalamus in pulses that are crucial for reproduction (Moenter, 2015). This pulsatile release can only be achieved if many GnRH-producing neurons are able to coordinate their activity to release the hormone at the same time, but it was not clear how this is achieved. Now, in eLife, Jian Qiu and colleagues – who are based at the Oregon Health and Science University and the University of Washington – report that neurons in the hypothalamus that produce a protein called kisspeptin can synchronize their activity and activate GnRH neurons (Qiu et al., 2016).

A previous study suggested that a group of kisspeptin-producing neurons in a brain region called the arcuate nucleus of the hypothalamus – called Kiss1^ARH^ neurons for short – might be responsible for generating the GnRH pulses (Okamura et al., 2013). However, there is also a non-pulsatile surge in GnRH release in females before they ovulate. This surge appears to be driven by other groups of kisspeptin neurons (referred to as Kiss1^AVPV/PeN^ neurons) in two other parts of the hypothalamus (Herbison, 2016). A recent tracing study suggests that Kiss1^ARH^ neurons do not have any direct contact with the cell bodies of GnRH neurons, but may instead be linked to them via Kiss1^AVPV/PeN^ neurons (Yip et al., 2015).

Qiu et al. used a technique called optogenetics to investigate how kisspeptin neurons control the release of GnRH in mice. Genetically modifying the mice to express a light-sensitive ion channel protein called channelrhodopsin in their Kiss1^ARH^ neurons allowed Qiu et al. to activate these neurons with beams of light. This “photostimulation” of Kiss1^ARH^ neurons produced electrical activity in these cells known as a slow excitatory post synaptic potential. This electrical activity seemed to depend on inputs from other Kiss1^ARH^ neurons and relied on two receptor proteins that detect the neurotransmitters neurokinin B and dynorphin, which are released by Kiss1^ARH^ neurons. Furthermore, the photostimulation of Kiss1^ARH^ neurons on one side of the brain produced slow excitatory post synaptic potentials in Kiss1^ARH^ neurons on the other side of the brain.

Further experiments revealed that photostimulating Kiss1^ARH^ neurons can produce activity in the GnRH neurons of mice. Drugs that activate a neurokinin B receptor protein on Kiss1^ARH^ neurons also have a similar effect in mouse brain slices, which suggests that Kiss1^ARH^ neurons activate each other to stimulate GnRH neurons. Qiu et al. also show that Kiss1^ARH^ neurons stimulate GnRH neurons by activating Kiss1^AVPV/PeN^ neurons (Figure 1).

**Figure 1.**
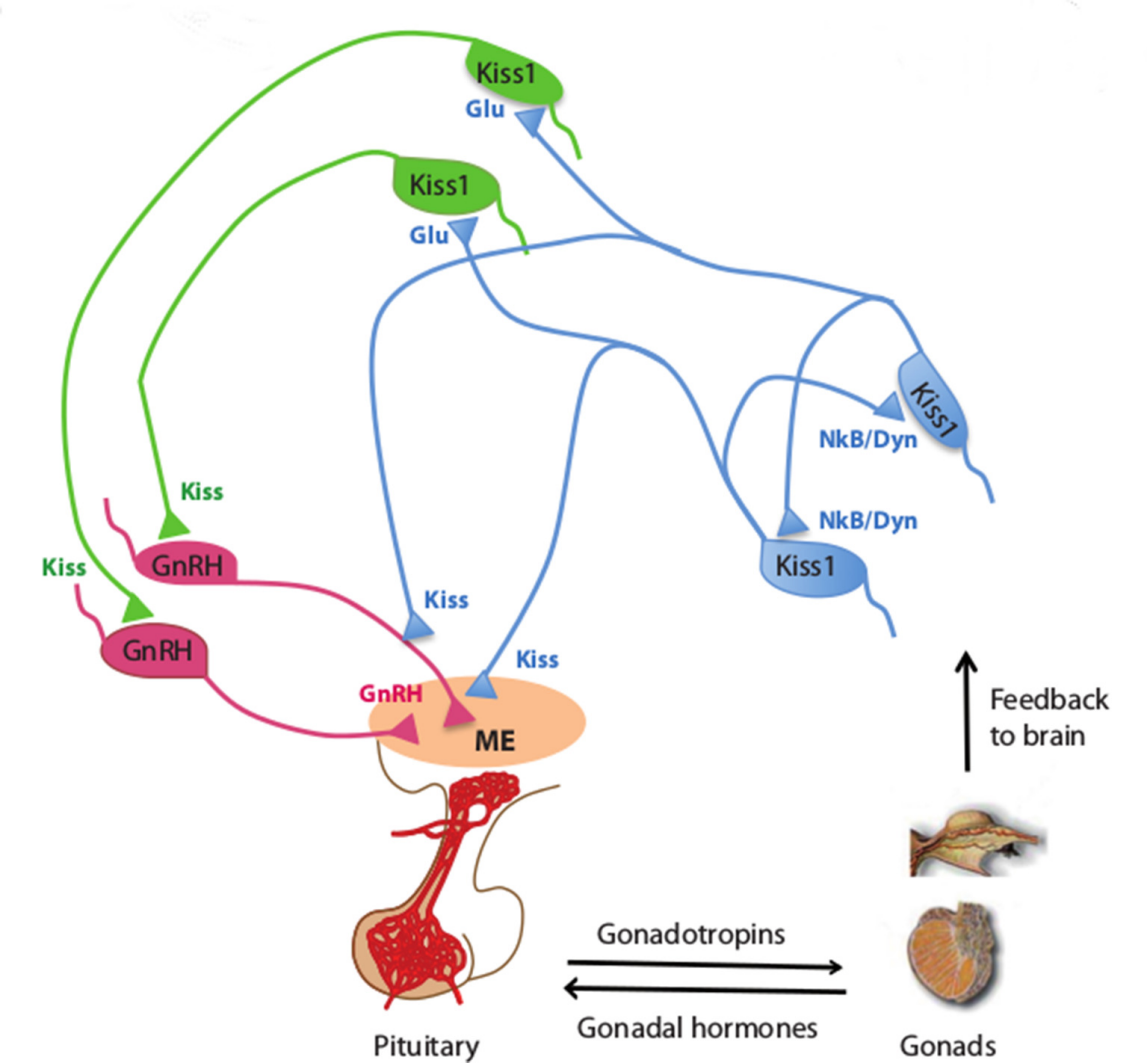
Kisspeptin neurons coordinate the release of hormones that regulate reproduction. In mammals, several hormones interact to regulate fertility and reproduction. Gonadotrophin releasing hormone (GnRH) is released by neurons (pink) in an area of the brain called the median eminence (ME; peach oval), which is part of the hypothalamus. This triggers the release of gonadotrophin and other hormones from the pituitary gland, which leads to the production of sex cells and sex (gonadal) hormones in the gonads. In turn, the gonads provide feedback to the system by regulating the release of GnRH (not shown) and gonadotrophin. Qiu et al. found that kisspeptin neurons in the hypothalamus synchronize their activity and drive GnRH neuronal activity. Kisspeptin neurons in the arcuate nucleus of the hypothalamus (Kiss^ARH^ neurons; blue) activate other Kiss^ARH^ neurons by releasing two neurotransmitters called neurokinin B (NkB) and dynorphin (Dyn). These neurons also activate kisspeptin neurons in the anteroventral periventricular nucleus and the periventricular preoptic nucleus (Kiss1^AVPV/PeN^ neurons; green) in a pathway that involves another neurotransmitter called glutamate (Glu). In turn, the Kiss1^AVPV/PeN^ neurons contact the cell body of GnRH neurons to stimulate the release of GnRH (Glanowska and Moenter, 2015). Kiss^ARH^ neurons can also directly contact the fibers of GnRH neurons in the arcuate nucleus (Ciofi et al., 2006), which has been proposed to stimulate GnRH release without involving the cell body of GnRH neurons.

Together, these findings suggest that Kiss1^ARH^ neurons on both sides of the brain coordinate their activity to stimulate the release of GnRH from the hypothalamus. Further work is needed to find out if this synchronization is sufficient to regulate the pulsatile release of GnRH. The photoactivation stimulus used in this study triggered very strong activity in the Kiss1^ARH^ neurons: are there any inputs to Kiss1^ARH^ neurons in normal mice that can trigger similarly high levels of activity? A future challenge is to investigate whether the kisspeptin neurons in the arcuate nucleus set the pattern of GnRH pulses, or whether they simply relay synchronized patterns that they receive from other neurons (Israel et al., 2014; Marder et al., 2014).

## References

[bib1] Boehm U, Bouloux PM, Dattani MT, de Roux N, Dodé C, Dunkel L, Dwyer AA, Giacobini P, Hardelin JP, Juul A, Maghnie M, Pitteloud N, Prevot V, Raivio T, Tena-Sempere M, Quinton R, Young J (2015). Expert consensus document: European Consensus Statement on congenital hypogonadotropic hypogonadism--pathogenesis, diagnosis and treatment. Nature Reviews Endocrinology.

[bib2] Cimino I, Casoni F, Liu X, Messina A, Parkash J, Jamin SP, Catteau-Jonard S, Collier F, Baroncini M, Dewailly D, Pigny P, Prescott M, Campbell R, Herbison AE, Prevot V, Giacobini P (2016). Novel role for anti-Müllerian hormone in the regulation of GnRH neuron excitability and hormone secretion. Nature Communications.

[bib3] Ciofi P, Leroy D, Tramu G (2006). Sexual dimorphism in the organization of the rat hypothalamic infundibular area. Neuroscience.

[bib4] Clarke SA, Dhillo WS (2016). Kisspeptin across the human lifespan:evidence from animal studies and beyond. Journal of Endocrinology.

[bib5] Glanowska KM, Moenter SM (2015). Differential regulation of GnRH secretion in the preoptic area (POA) and the median eminence (ME) in male mice. Endocrinology.

[bib6] Herbison AE (2016). Control of puberty onset and fertility by gonadotropin-releasing hormone neurons. Nature Reviews Endocrinology.

[bib7] Israel JM, Cabelguen JM, Le Masson G, Oliet SH, Ciofi P (2014). Neonatal testosterone suppresses a neuroendocrine pulse generator required for reproduction. Nature Communications.

[bib8] Marder E, O'Leary T, Shruti S (2014). Neuromodulation of circuits with variable parameters: single neurons and small circuits reveal principles of state-dependent and robust neuromodulation. Annual Review of Neuroscience.

[bib9] Moenter SM (2015). Leap of Faith: Does Serum Luteinizing Hormone Always Accurately Reflect Central Reproductive Neuroendocrine Activity?. Neuroendocrinology.

[bib10] Okamura H, Tsukamura H, Ohkura S, Uenoyama Y, Wakabayashi Y, Maeda K (2013). Kisspeptin and GnRH pulse generation. Advances in Experimental Medicine and Biology.

[bib11] Qiu J, Nestor CC, Zhang C, Padilla SL, Palmiter RD, Kelly MJ, Ronnekleiv OK (2016). High frequency stimulation-induced peptide release synchronizes arcuate kisspeptin neurons and excites GnRH neurons. eLife.

[bib12] Yip SH, Boehm U, Herbison AE, Campbell RE (2015). Conditional Viral Tract Tracing Delineates the Projections of the Distinct Kisspeptin Neuron Populations to Gonadotropin-Releasing Hormone (GnRH) Neurons in the Mouse. Endocrinology.

